# Exploring the influence of microgravity on chemotherapeutic drug response in cancer: Unveiling new perspectives

**DOI:** 10.1111/jcmm.18347

**Published:** 2024-05-02

**Authors:** Preksha Manish Vora, Sudharshan Prabhu

**Affiliations:** ^1^ Department of Cell and Molecular Biology, Manipal School of Life Sciences Manipal Academy of Higher Education Manipal India

**Keywords:** cancer progression, chemotherapeutic drugs, microgravity, space medicine

## Abstract

Microgravity, an altered gravity condition prevailing in space, has been reported to have a profound impact on human health. Researchers are very keen to comprehensively investigate the impact of microgravity and its intricate involvement in inducing physiological changes. Evidenced transformations were observed in the internal architecture including cytoskeletal organization and cell membrane morphology. These alterations can significantly influence cellular function, signalling pathways and overall cellular behaviour. Further, microgravity has been reported to alter in the expression profile of genes and metabolic pathways related to cellular processes, signalling cascades and structural proteins in cancer cells contributing to the overall changes in the cellular architecture. To investigate the effect of microgravity on cellular and molecular levels numerous ground‐based simulation systems employing both in vitro and in vivo models are used. Recently, researchers have explored the possibility of leveraging microgravity to potentially modulate cancer cells against chemotherapy. These findings hold promise for both understanding fundamental processes and could potentially lead to the development of more effective, personalized and innovative approaches in therapeutic advancements against cancer.

## 1 INTRODUCTION

Gravity and electromagnetic forces play a pivotal role in influencing the course of biological evolution on Earth. These forces have a significant impact on organisms, influencing their structure, function and behaviour.^1^ Extensive evidence indicates that physical forces play a crucial role in regulating a wide range of cellular activities, encompassing cell behaviour, function and communication.^2^ Gravity, in particular, plays a critical role in cellular physiology and behaviour. Humans and other organisms on Earth have adapted to Earth's gravity of ~9.907 m/s^2^, which exerts mechanical stresses crucial for normal body functions and maintaining homeostasis.^3^

In space, the gravitational force is very low or reduced and is termed microgravity. Microgravity has been reported to impact biological processes significantly. Cells exposed to microgravity undergo changes in their morphology, structure and genetic expression. This altered environment affects crucial cellular processes, including division, differentiation, signalling, protein synthesis, DNA repair and immune response.^2^ Space travellers experience microgravity during space exploration programmes, which significantly impacts the immune system, muscle and bone density and tissue repair.^4^, ^5^, ^6^ Gaining a comprehensive understanding of the cellular response under microgravity holds immense potential for various applications, including cancer biology and potentially leading to the discovery of novel approaches for cancer treatment.

Cancer, a highly intricate and diverse disease condition characterized by the uncontrolled proliferation and division of abnormal cells.^7^ It exhibits distinct characteristics such as autonomous growth, resistance to the signals that govern cell division, unrestrained replication, resistance to apoptosis, continuous angiogenesis and ability to invade and metastasize to distant tissues.^8^

Recent studies have investigated the effect of microgravity conditions on the growth and progression of cancer cells.^2^ in vitro studies utilizing ground‐based simulation systems have highlighted the impact of microgravity on cancer cells, demonstrating altered metastatic potential in various cancer types including lung cancer and melanoma cells. Lung cancer cells exposed to simulated microgravity (SMG) exhibited decreased gene expression, resulting in a decrease in their ability to metastasize.^9^, ^10^

Chemotherapy is a common modality used to target and treat cancer; however, cancer metastasis is the key cause of cancer therapy failure, leading to death in over 90% of cancer cases.^11^ Metastasis, a multifaceted process, encompasses the dissemination of cancer cells from their primary location to distant regions of the body. It occurs across different cancer types, despite the vast diversity in their molecular biology, development and prognosis.^12^

This review delves into the impact of microgravity on the efficacy of chemotherapy and its effect on cancer cells. By exploring the microgravity influence on the efficacy of anticancer treatment, this report offers insights crucial for optimizing the use of chemotherapeutics agents and developing more effective strategies for combating cancer.

## 2 APPROACHES TO SIMULATING MICROGRAVITY

To study the impact of microgravity on biological systems, researchers have employed ground‐based simulation systems that replicate space‐like microgravity conditions. These simulation systems provide valuable insights into gravity‐dependent phenomena using a wide range of organisms. However, each simulator has its own limitations and potential artefacts, which can inadvertently introduce unintended effects and distort the desired microgravity response.^13^ Consequently, the misinterpretation of responses to these side effects as specific microgravity effects can occur. Furthermore, when comparing results from experiments conducted on different simulators, inconsistencies or even conflicting responses may arise. Hence, it is crucial to critically evaluate the physical parameters and principles of each simulator as well as their specific impact on biological processes and organisms of varying sizes. Not all simulators and operational modes are equally suitable for accurately simulating microgravity across all processes and organisms. Simulation of microgravity can be performed for both in vitro and in vivo studies to understand microgravity‐associated alterations at the cellular level.

### 2.1 Simulated microgravity (SMG) for in vitro systems

#### 2.1.1 Free fall machine (FFM)

**FIGURE 1:**
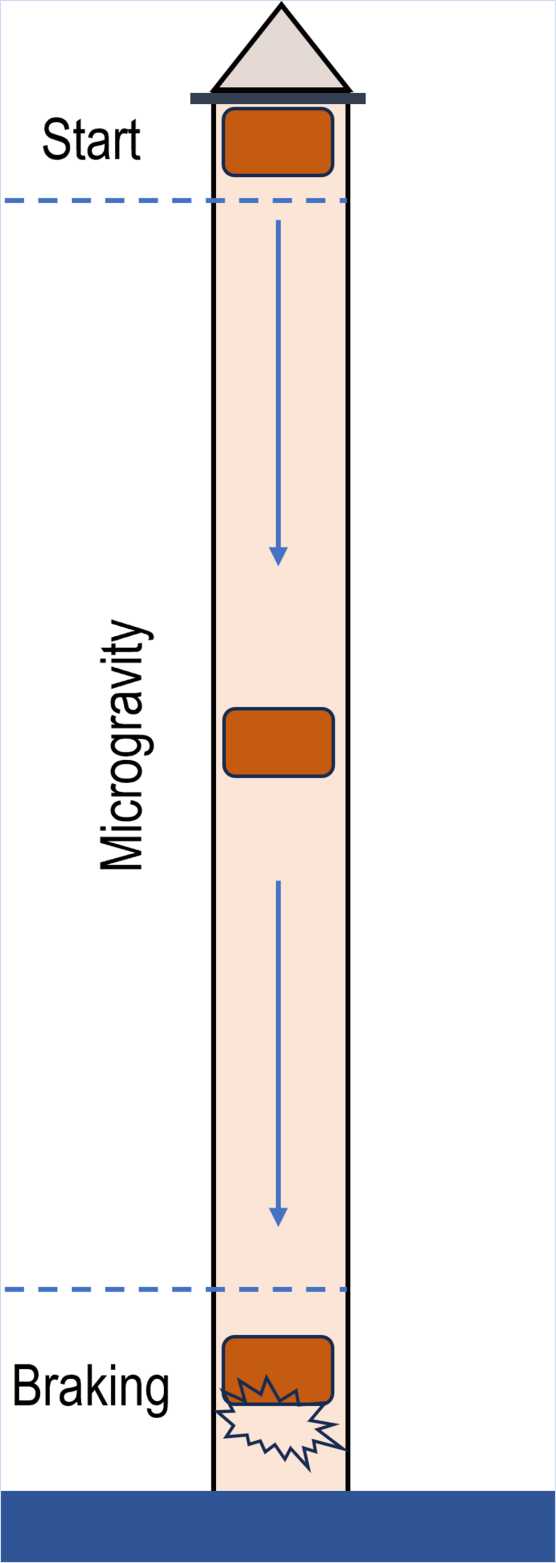
Schematic representation of a free fall machine (FFM).

In FFM (Figure 1), the sample is passed through an elongated tube, which ensures free fall of the sample for a few milliseconds before being acted upon by an air current that launches the sample upwards. The FFM creates a state of weightlessness as the sample attains terminal velocity during free fall, causing the sample (cells) to not experience gravitational force, resulting in weightlessness.^14^, ^15^

#### 2.1.2 Clinostats

**FIGURE 2:**
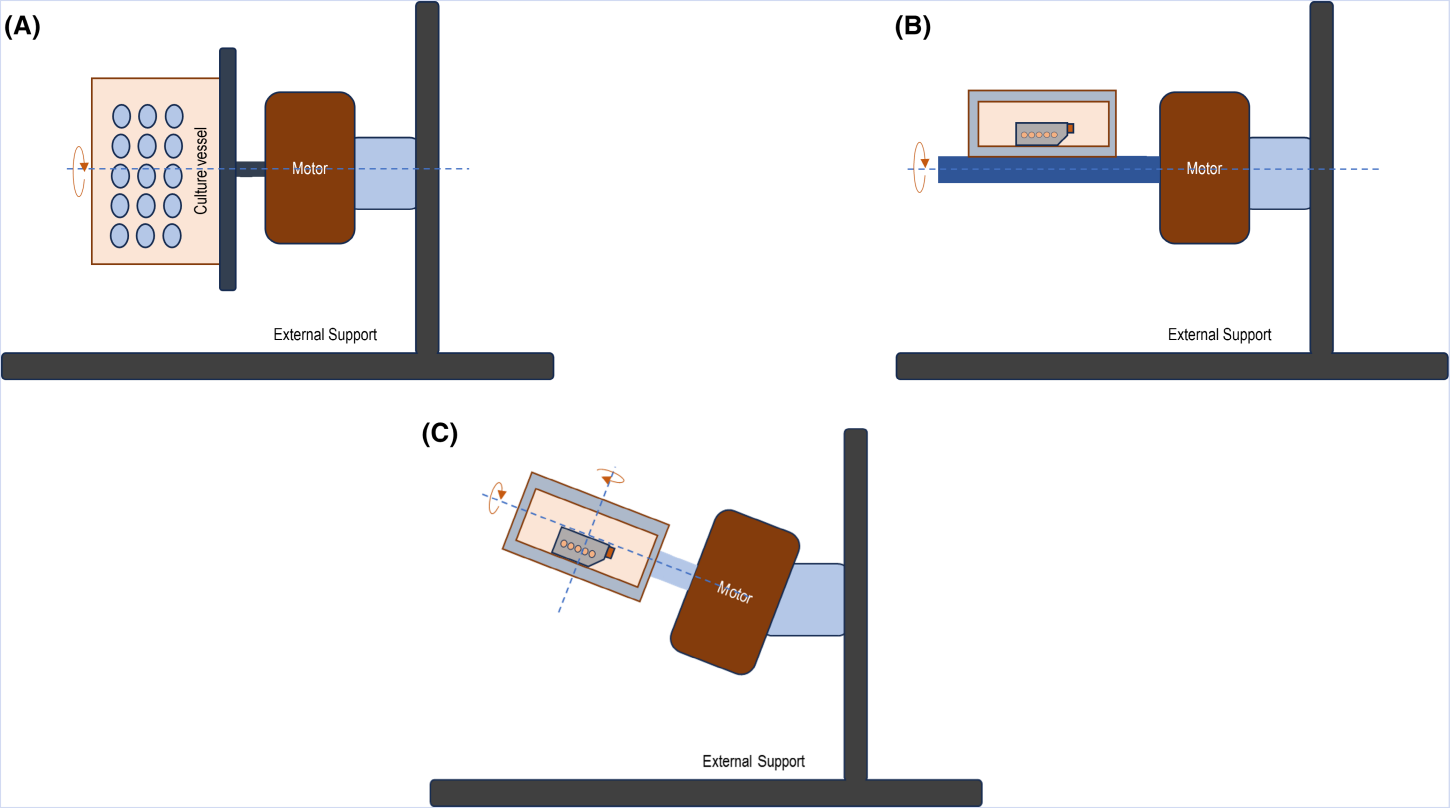
Schematic representation of (A) 1D clinostat, (B) 2D clinostat and (C) 3D clinostat.

Clinostats, also referred to as random positioning machine (RPM), is a commonly utilized equipment to simulate microgravity.^16^ Clinostats rely on the principle of clinorotation wherein centrifugal forces are generated whose intensities depend upon the distance of the sample (cells) from the rotation axes of the clinostat.^17^ Furthermore, based on the number of rotation axes, clinostats can be categorized into 1D/2D and 3D (three‐dimensional) systems. In 1D system (Figure 2A), the sample is rotated in one axis (vertical axis). In this position, the object experiences a gravity pull towards its lower side.^18^ In a 2D clinostat (Figure 2B), the sample (cells) is rotated about the horizontal axis via a rotary device in a continuous fashion, causing the gravity vector to change with respect to the constantly changing direction of the sample in rotation.^19^ By selecting an optimal rotational speed (10–20 rpm) with minimal centrifugal force, the sample experiences a microgravity environment.^20^ In the 3D clinostat (Figure 2C), the frames operate at constant speed and direction and consist of two perpendicular frames (gimbal‐mount), one inside the other. These frames can rotate independently.^21^ The orientation of the gravity vector is continuously altered, resulting in an averaged gravity vector that mimics a microgravity environment.^22^

#### 2.1.3 Rotary cell culture system (RCCS)

**FIGURE 3:**
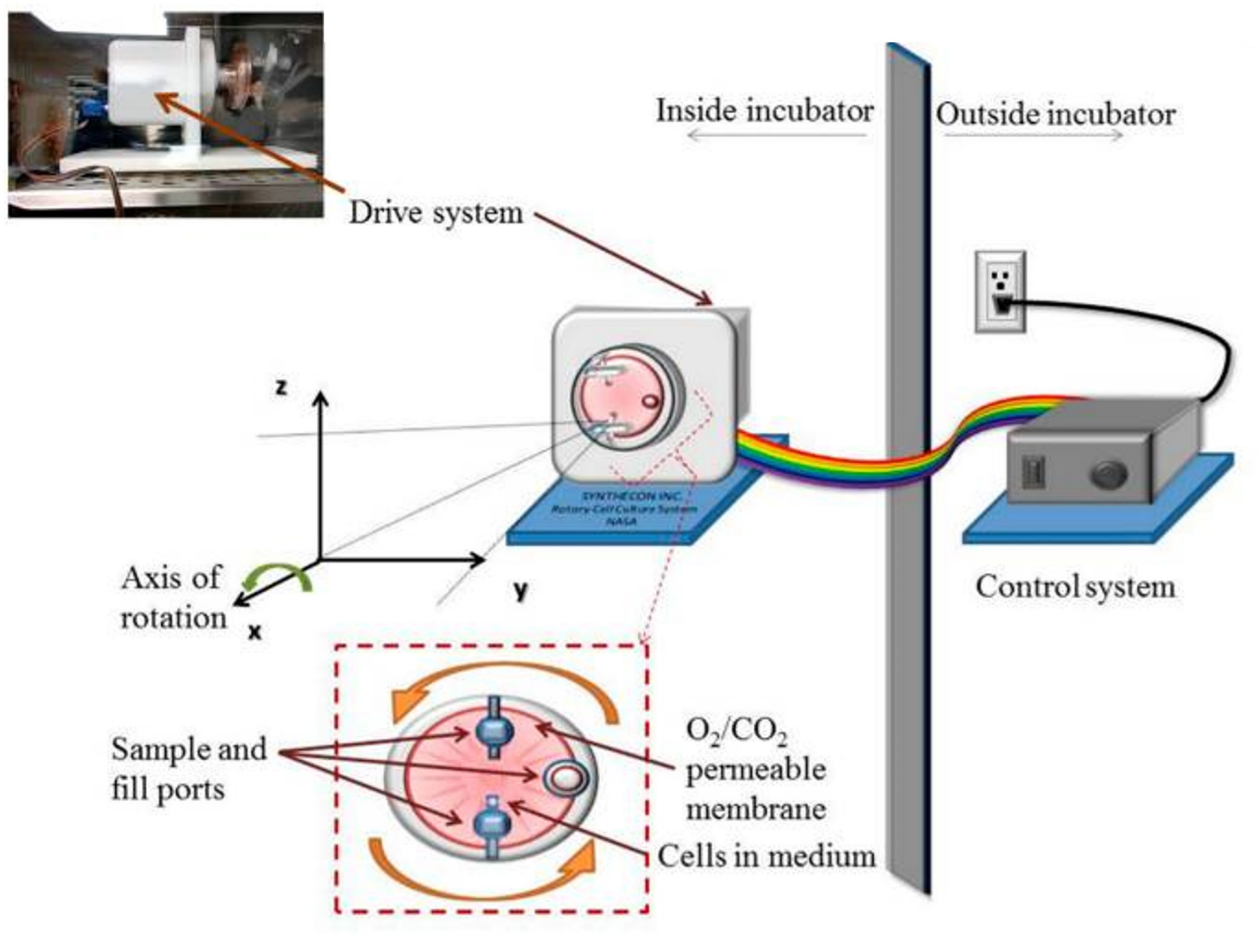
Rotary cell culture system (RCCS) placed inside a CO_2_ incubator.^1^

RCCS (Figure 3) is a novel technique designed for the cultivation of anchorage‐dependent or suspension cells. The system consists of horizontally rotated culture vessels, which provide an environment for cell growth. During the culture process, the rotation speed can be customized to counteract cell sedimentation. The RCCS provides a unique setting with low shear forces, efficient mass transfer and SMG conditions.^23^

#### 2.1.4 Diamagnetic levitation

**FIGURE 4:**
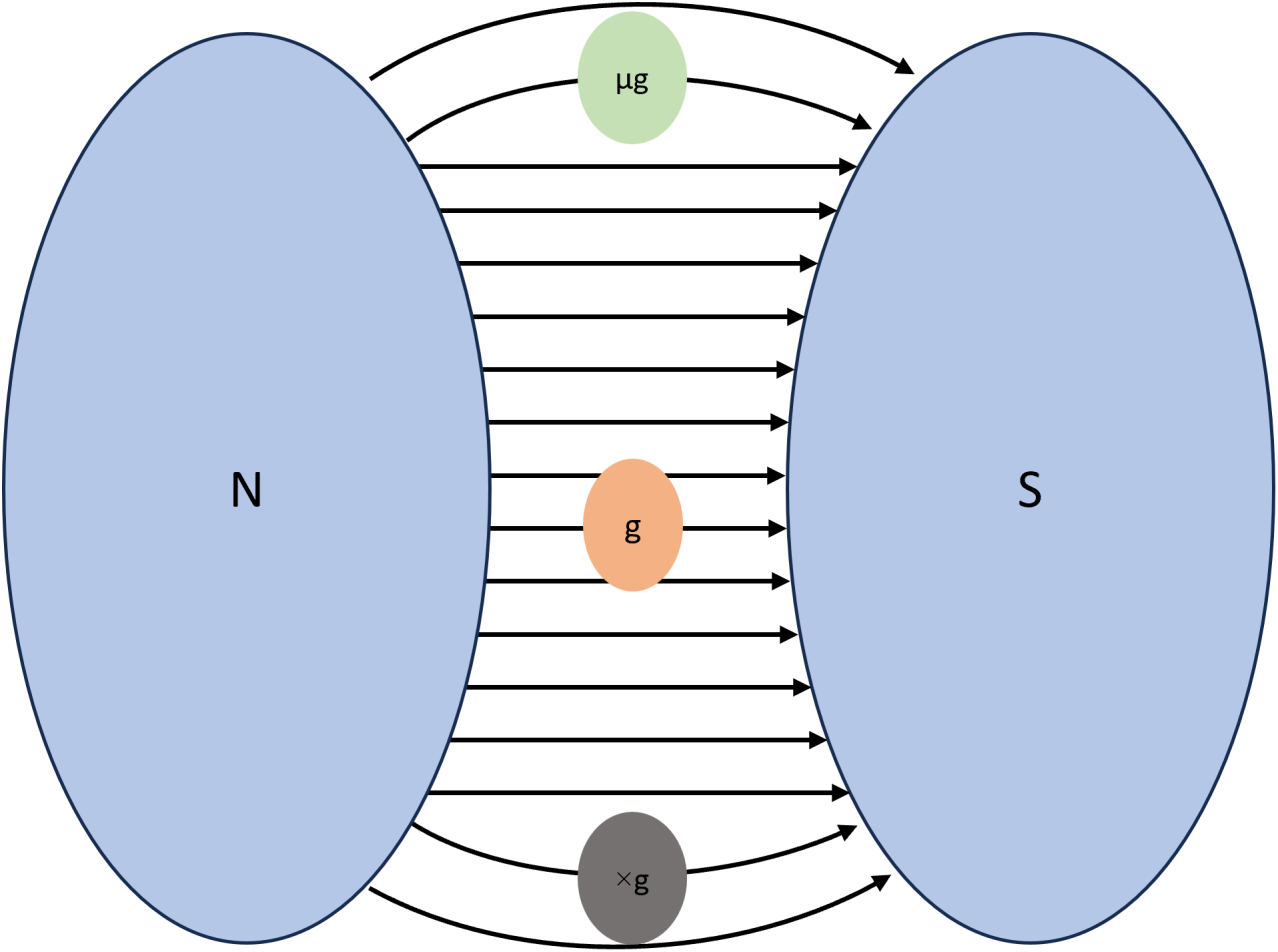
Schematic representation of diamagnetic levitation.

Diamagnetic levitation (Figure 4) employs a magnetic adjustable gravity simulator, which employs an intense magnetic field to generate forces that oppose the gravity force. Diamagnetic materials such as water and organic materials are repelled upwards or downwards depending on their position in the magnet. By regulating the magnetic field in the system microgravity conditions can be simulated. Levitation is achieved when the magnetic forces balance the weight of the material and create a weightless environment by reducing internal gravity‐induced stress.^24^ This technique can simulate partial gravity by placing samples at different points in the magnetic field gradient and produce intermediate gravitational forces similar to those of the space environment.^25^ Diamagnetic levitation is extensively used to investigate the effect of microgravity in vitro experiments.

### 2.2 Simulated microgravity for in vivo systems

**FIGURE 5:**
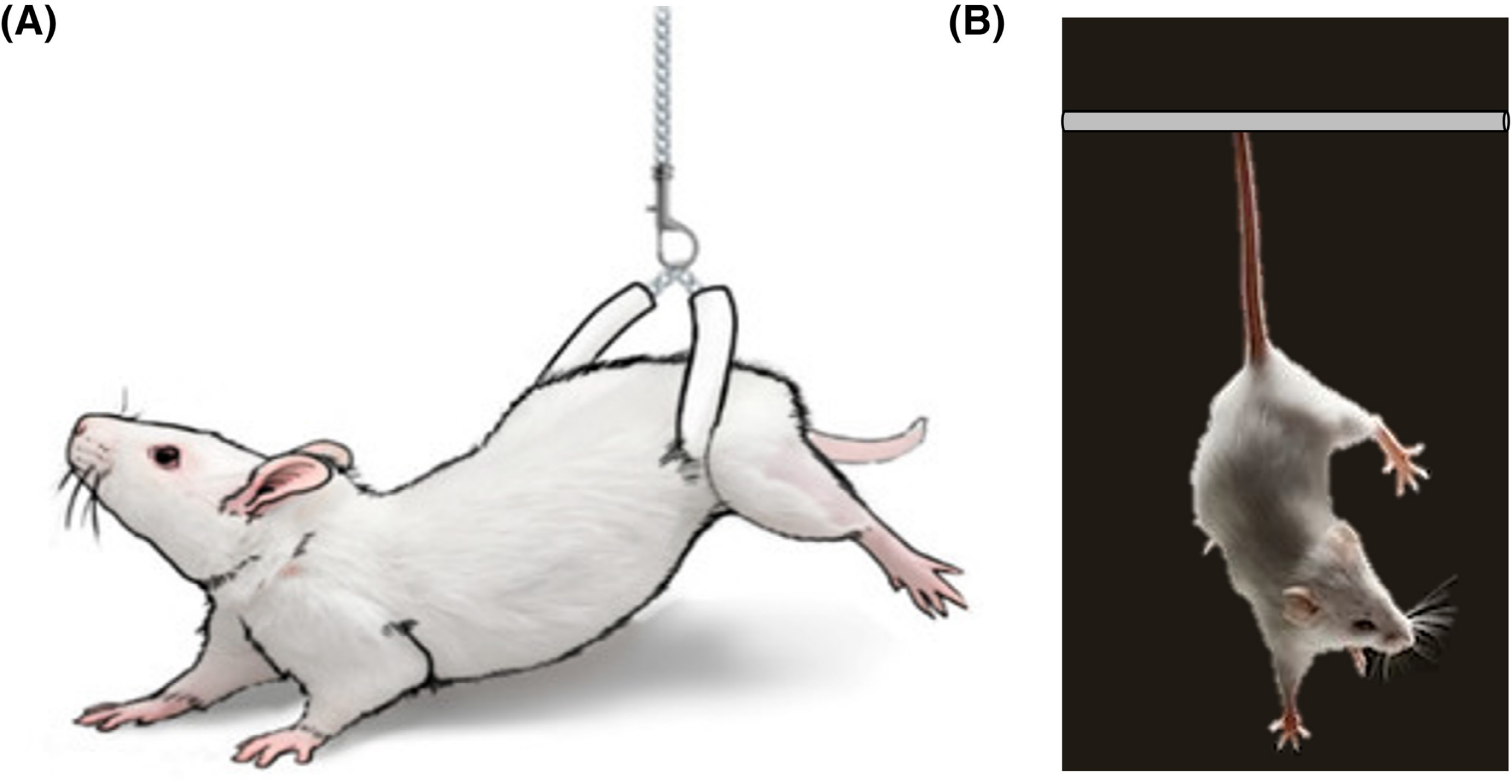
Schematic representation of (A) hindlimb unloading (B) tail suspension for rodent models.^28^

To investigate the impact of microgravity on physiological processes, cellular and biochemical parameters in in vivo systems, researchers employ ground‐based simulation techniques. Currently, researchers employ hindlimb unloading and tail suspension models (Figure 5A and Figure 5B) to study the impact of microgravity on mice and rat models. In these models, the body of the animal makes an approximately 30° angle from the floor of the cage such that the animal does not touch the grid floor with its back feet.^26^, ^27^ This suspension ensures the removal of mechanical loading from the hindlimbs simulating microgravity conditions.^28^

### 2.3 Simulation of microgravity in humans

#### 2.3.1 Head‐down tilt method

**FIGURE 6:**
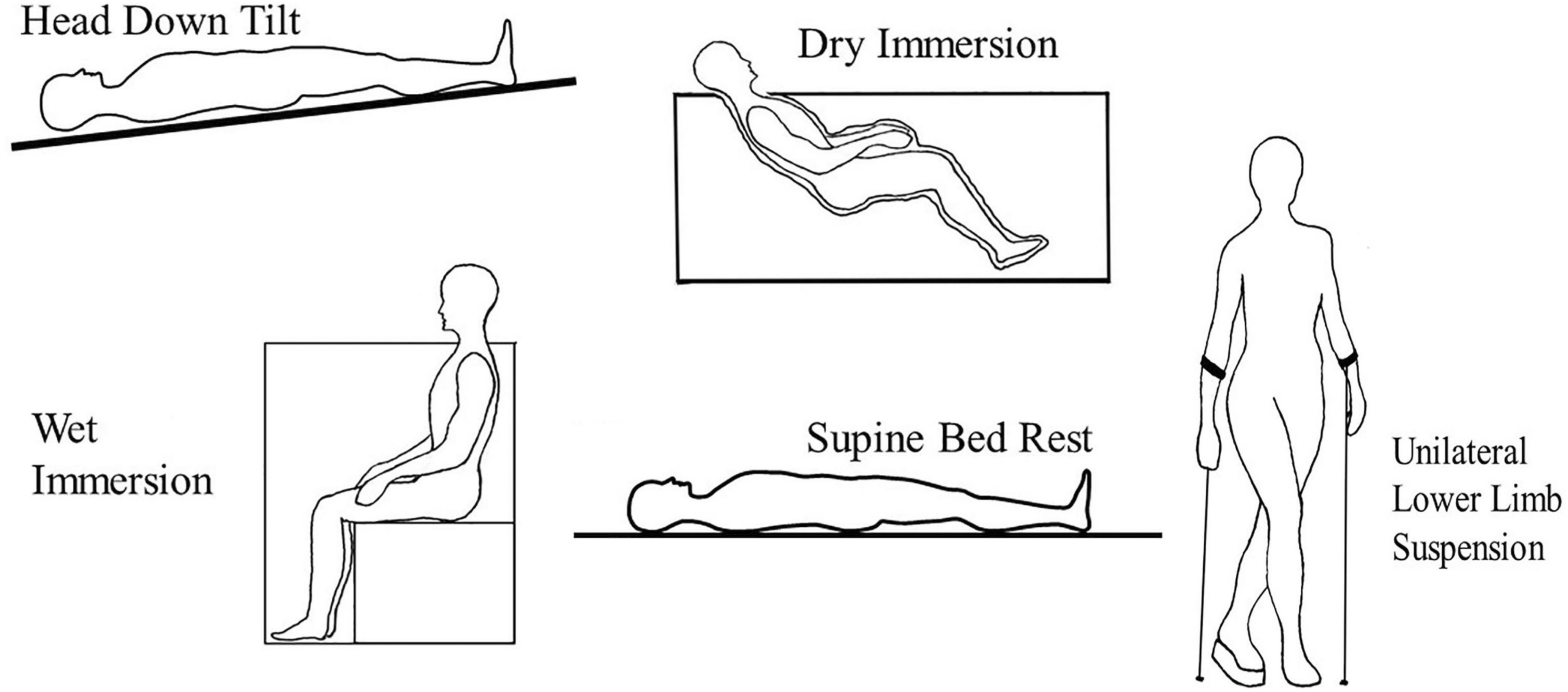
Schematic representation of microgravity simulation methods in humans.^29^

In the HDT method (Figure 6) the subjects are positioned in a recline position on an inclined bed tilted at a 6° angle with the head closer to the ground and feet elevated. HDT induces a headward fluid shift of blood from the lower body to the upper body causing hydrostatic pressure in blood vessels.^29^ HDT methods are best employed in studying various physiological systems, musculoskeletal and cardiovascular disorders.^30^ HDT methods can provide valuable insights into understanding the effect of SMG.

#### 2.3.2 Dry immersion method

The dry immersion method is another widely used ground‐based model for studying the effect of microgravity (Figure 6). In the dry immersion method, the subjects are submerged in water up to the neck, inducing a floating sensation that stimulates the microgravity effect.^31^ Dry immersion also induces a headward fluid shift, similar to the condition experienced in space. Dry immersion offers a valuable method for studying the physiological responses to microgravity, however, its limitations should be considered when interpreting the results.^29^, ^31^

#### 2.3.3 Wet Immersion method

The water immersion method involves submersion into water from the neck down (Figure 6).^32^ In the wet immersion method, the subjects are immersed in water, which exerts hydrostatic pressure causing redistribution of body fluids towards the upper body, simulating the fluid shifts that occur in microgravity.^33^

#### 2.3.4 Unilateral lower limb suspension (ULLS) method

ULLS is a cost‐effective method used to study the effect of microgravity on the human body (Figure 6). It involves elevating one leg using a tall boot and crutches such that one foot is not in contact with the ground and allowing the subject to maintain mobility.^27^ In the ULLS method, the hydrostatic pressure is absent. This method provides valuable insights into localized musculoskeletal adaptations similar to those observed during space travel.^34^ However, ULLS is not ideal for evaluating the global effect and long‐term impact of microgravity.^29^

#### 2.3.5 Supine bed rest method

In the supine bed rest method, the subjects lie in a supine position for an extended period of time (Figure 6). This method simulates some of the physiological changes that occur during spaceflight or extended duration of inactivity, such as reduced physical activity and fluid distribution.^30^ The supine bed rest model allows the researcher to investigate the effect of prolonged immobility on various physiological systems including alterations in metabolic processes and musculoskeletal changes.^35^ By comparing the results of subjects exposed to supine bed rest to those in the space environment, researchers can gain insight into the specific effect of microgravity on the body.^29^

## 3 CANCER PROGRESSION

Cancer metastasis can occur via various modes that involve epithelial–mesenchymal transition (EMT),^36^ accumulation of mutations in stem cells^37^ and tumour‐associated macrophages promoting metastasis progression.^38^ Genetic mutations play a critical role in the development and progression of metastatic cancer. Studies have reported that mutations in genes such as PTEN (Phosphatase and TENsin homologue deleted on chromosome 10), CDKN2A (cyclin‐dependent kinase inhibitor 2A), TP53, KRAS (K‐Ras), SMAD4 (Mothers against decapentaplegic homologue 4) and BRCA1/2 (Breast cancer 1 and 2) have been associated with metastatic cancer.^39^

**FIGURE 7:**
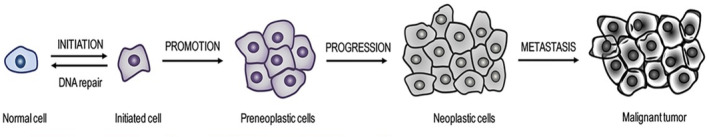
Stages of tumour development.^97^

The development of cancer involves four stages initiation, promotion, progression and metastasis, each marked by distinct molecular and cellular alterations (Figure 7). The initiation phase is a critical phase that involves genetic alterations resulting in the imbalance of biochemical signalling pathways that regulate cellular processes leading to pre‐cancerous cells.^40^ In the promotion phase, the pre‐cancerous cells are stimulated by promoting factors to create a favourable environment to develop into preneoplastic lesions or benign tumours leading to malignant tumours.^41^ Malignant tumours evade normal cellular control and instigate invasion through the degradation of extracellular matrix (ECM) by matrix metalloproteins (MMPs). The process of tumour invasion is further driven by cytokines such as interleukins (IL‐6 and IL‐8), genetic alteration in the tumour suppression genes (ras family and p53 tumour suppressors genes) and compromised DNA repair machinery.^8^, ^42^, ^43^ Another critical factor is angiogenesis, driven by hypoxia‐inducible factor (HIF) which stimulates vascular endothelial growth factor (VEGF) and promotes cancer progression.^44^

The final stage of cancer development is metastasis, where the cells disseminate from the site of origin to distant organs.^45^ This process is orchestrated by a complex network of pathways and factors that regulate gene expression and cellular behaviour.^46^ One crucial aspect is the activation of EMT,^47^ EMT activation plays a critical role in developing resistance and understanding the role of EMT is pivotal for developing effective cancer treatments.^48^, ^49^, ^50^

## 4 CURRENT TREATMENT MODALITIES

Advancements in technology have revolutionized cancer treatments and deepened our understanding of the biological processes involved. Various conventional and advanced methods are used to combat cancer.^51^ Treatment modalities have improved for better precision and effectiveness, leading to improved survival rates and enhanced quality of life of the patients. The treatment modalities include surgery, chemotherapy, radiation therapy, hormonal therapy, targeted therapy and more.^52^ Despite the significant progress in cancer treatment, chemotherapy continues to play a central role as a prominent alternative for advanced‐stage malignancies where surgery or radiation may not be suitable due to specific circumstances.^53^ However, drug resistance remains a major challenge in cancer treatment, leading to increased instances of relapse and poor survival.^54^

### 4.1 Chemotherapy

Chemotherapy is a systemic approach widely employed for the treatment of malignant tumours, utilizing cytostatic drugs to target and halt the growth of cancer cells throughout the body for various cancers.^55^ This approach was inspired by the use of nitrogen mustard, a potent DNA alkylating agent.^56^ Currently, chemotherapy employs a diverse range of anticancer drugs with distinct mechanisms of action, inducing apoptosis by inhibiting cell division and precisely targeting cancer cells.^57^ Chemotherapy offers versatile options for cancer treatment, serving as a standalone therapy or synergizing with other oncological treatments in comprehensive approaches.^55^ The first is neoadjuvant chemotherapy, which is used to reduce the tumour size before surgery or radiation therapy and the second is adjuvant chemotherapy, which is used to destroy remnant cancer cells after surgery or radiation therapy.^58^, ^59^

Chemotherapy drugs are classified as alkylating agents, antimetabolites, alkaloids, platinum derivatives and other natural products.^60^ The effect of chemotherapy is intricately linked to the cell cycle of cancer cells. The cytostatic drugs primarily target cells actively progressing through the cell cycle, disrupting the transition between different phases.^53^

### 4.2 Challenges in chemotherapy

While chemotherapy has demonstrated effectiveness in slowing tumour progression, it comes with a significant drawback: its impact on both cancerous and healthy cells leads to toxicity.^61^ Additionally, another substantial challenge is the emergence of resistance to chemotherapeutic drugs.^62^ Despite significant advancements in cancer treatment, overcoming resistance to chemotherapeutic drugs remains a major drawback. This adverse effect plays a pivotal role in disease relapse and contributes to poor survival rates in patients.^54^ Chemotherapeutic drug resistance can occur either by intrinsic or acquired mechanisms. Intrinsic mechanism is when cells acquire resistance against the drug at the onset of treatment. On the other hand, acquired mechanisms occur much later, during the course of cancer treatment.^63^ Emerging evidence indicates that microgravity can effectively modulate various cellular processes, including cytoskeletal alteration, reduced cell proliferation, altered gene expression and reduced invasion and metastasis.^64^

## 5 EFFECT OF MICROGRAVITY ON CANCER PROGRESSION

The cytoskeleton architecture plays an indispensable role in determining cell shape, facilitating cell migration and controlling cell survival and proliferation.^65^ The cytoskeletal protein consists of actin, intermediate filaments and microtubules which influence the cancer cell behaviour and contribute to the cellular processes critical for malignancy.^64^, ^65^ Microgravity induces profound changes in the cytoskeleton protein leading to the activation of numerous genes that trigger diverse biochemical pathways.^66^, ^67^, ^68^, ^69^

This intricate interplay between gravitational forces and the cytoskeleton plays a pivotal role in influencing essential cell functions such as proliferation, differentiation, remodelling of extracellular matrix and apoptosis.^70^ Cancer cells on exposure to microgravity form spheroids due to increased expression of actin, intermediate filaments and extracellular matrix components such as laminins, fibronectins, collagen and chondroitin sulphate.^71^, ^72^ Studies have reported that reorganization and restructuring of actin filaments is very critical for cancer metastasis.^73^

To understand the impact of microgravity on structural and functional aspects of cells, Vassey et al.^74^ investigated the effect of using mammary cancer cells (MCF‐7), the results showed notable alteration in the nuclear proteins (Ki‐67), a cell proliferation marker resulting in extended mitotic phase. Further, the cells also showed alteration in actin filaments and phosphotyrosine signal transduction. The MCF‐7 cells also showed modification in the DNA distribution in the interphase cells, indicating the changes in the chromatin structure. All the effects have shown a profound influence on cell cycling, cytoskeletal dynamics, chromatin structure and gene expression.^74^

Migration and metastasis of the cancer cells are majorly associated with the modulation of ECM components particularly Matrix metalloproteinases (MMPs) and TIMPs (Tissue inhibitors of metalloproteinase).^75^, ^76^, ^77^ MMPs are majorly involved in the degradation of ECM and TIMPs play a crucial role in regulating ECM by MMPs, imbalance in these enzymes can significantly promote cancer metastasis.^78^, ^79^, ^80^ Recently, Ahn et al.^81^ investigated the effect of microgravity on the proliferation and migration of non‐small‐cell lung cancer (A549 and H1703). In both A549 and H1703 cells exposed to microgravity for 24 and 48 hours, the expression levels of MMP‐2, MMP‐9, TIMP‐1 and TIMP‐2 were enhanced. The findings indicate that microgravity promotes cell migration by modulating the ECM components. However, the migration and metastasis varies across different types of cancer.^81^

Long‐term exposure to microgravity has a significant impact on signal transduction along the cytoskeleton to the nucleus and induces alteration in ECM proteins.^82^ Infanger and team^82^ investigate the effect of the long‐term influence of microgravity on papillary thyroid cancer cells (ONCO‐DG‐1) by exposing cells to the random positioning machine for 120 hours. The papillary thyroid cancer exhibited early alteration in the cytoskeleton and ECM proteins, triggering the formation of spheroids. Furthermore, enhanced accumulation of ECM components such as collagen type I and II, osteopontin, chondroitin sulfate, vimentin and fibronectin were observed. The levels of transforming growth factor beta‐1 (TGF‐β1) and its receptor (TGFBR2) were upregulated from 24 h until 120 h clinorotation, potentially contributing to ECM enhancement. TGF‐β1 plays a significant role in cancer cell progression, exhibiting a dual role in both suppression and metastasis of cancer. In the early onset of the malignancy, TGF‐β1 acts as a tumour suppressor and triggers apoptosis. However, in the advanced stage of malignancy, TGF‐β1 promotes EMT invasion and metastasis.^83^ These results suggest that microgravity can potentially alter the TGF‐β1 signalling pathway, leading to the suppression or metastasis of cancer.^84^

The impact of microgravity on poorly differentiated follicular thyroid cancer cells (TC) was determined by exposure to SMG in the CellBox‐1 study and real microgravity in the International Space Station (ISS) during the CellBox‐2 mission.^85^, ^86^ The findings of the study revealed that TC cells showed distinct cell phenotypes with cells showing spheroid formation in response to microgravity. The results suggested that gravitational unloading in microgravity impacted various cancer cell processes, such as differentiation, proliferation, apoptosis, growth and invasion. Gene expression studies indicated that significant upregulation of ECM protein genes like COL1A1, potentially linked to cell detachment and spheroid formation, while downregulation of β1‐integrin (ITGB1) suggested inhibition of cell adhesion. Altered gene expressions of cell adhesion factors (CAV1, ICAM1), growth factors (EGF, VEGFD) and inflammatory cytokines (IL‐6, IL‐8) were observed in space samples. Proto‐oncogene Src (SRC) and focal adhesion molecule, vinculin (VCL) were downregulated, along with clear downregulation of key signalling pathways (NF‐κB, ERK1/2).^87^ These interactions highlighted complex signalling relationships, indicating suppression of genes associated with growth, differentiation, proliferation, focal adhesion, progression and metastasis in spaceflight samples. Overall, the study suggests that microgravity induces a redifferentiation of thyroid cancer cells and a shift towards a less‐aggressive growth behaviour.

Cancer cell survival and metastasis majorly involve AKT activation.^88^ PTEN (Phosphatase and Tensin Homologue) suppresses AKT activation, preventing its nuclear translocation and further activating FOXO3 (Forkhead Box O3), resulting in tumour suppression.^88^ Recently, Arun and colleagues investigated the role of microgravity on PTEN/FOXO3/AKT pathway in colorectal cancer cells (DLD1, HCT116 and SW640).^89^ The results showed that all the CRC cells formed spheroids in response to microgravity, with distinct viability rates. Further, these cells showed distinct viability in response to microgravity. SW620 and HCT116 cells showed 20% viability, while DLD1 cells showed 40% viability under microgravity conditions. Gene expression studies indicated that cell death in response to microgravity was due to the upregulation of the tumour suppressors PTEN and FOXO3. This further led to the downregulation of AKT, triggering apoptosis by upregulation of CDKN2B and CDKN2D (CDK inhibitors).^89^ This study clearly indicates that microgravity regulates cell function by PTEN/FOXO3/AKT pathway.

To understand the dysregulation in gene networks associated with the cell cycle, oncogenes and cancer progression triggered by microgravity, a genome‐wide expression analysis was carried out in colorectal cancer cells (DLD‐1) and lymphoblast leukaemic cells (MOULT‐4).^90^ The findings revealed significant alterations in gene expression in response to microgravity. Specifically, DLD‐1 cells exhibited upregulation of 1801 genes and downregulated 2542 genes, whereas MOULT‐4 cells showed upregulation of 349 genes and downregulation of 444 genes. These changes induce alterations in the cytoskeleton, plasma membrane and intracellular signalling. Additionally, the study reported the dysregulation of the microRNA host genome, the microRNA‐22 known for its tumour suppressor activity, displayed significant upregulation during microgravity exposure, potentially contributing to anti‐proliferative effect.^90^ The migration and invasion of cancer cells is majorly regulated by Calcium ions (Ca^2+^).^91^ Intracellular Ca^2+^ plays a critical role in the reorganization of cytoskeletal proteins. Numerous calcium channels contribute to the invasion and migration of cancer cells, particularly store‐operated calcium entry (SOCE), which has been reported to be closely linked to the invasion and migration of cancer cells.^92^ Recent study by Shi and team reported that microgravity significantly reduced the invasive and migratory capabilities of glioblastoma (U87 cells) by lowering the expression of calcium ion regulatory proteins such as ORA11, thereby inhibiting SOCE, resulting in a reduced influx of Ca^2+^ into the cells.^93^ The findings of this study provide valuable insights into the relationship between microgravity and Ca^2+^ ions and their impact on cellular processes.

The proteins mammalian target of rapamycin complex 1 (mTORC1), focal adhesion kinase (FAK) and ras homologue gene‐family member A (RhoA) play a central role in maintaining cellular homeostasis. A recent study by Tan et al.^10^ has reported that microgravity can significantly downregulate key signalling molecules, such as FAK, RhoA and mTORC1, in melanoma cells (BL6‐10) which are involved in cell adhesion and migration.^10^ Further, the study also reported that microgravity activates Unc‐51‐like autophagy activating kinase 1 (ULK1) and AMP‐activated protein kinase (AMPK) resulting in reduced proliferation and metastasis of melanoma.

Autophagy is a catabolic process that degrades cytoplasmic components in response to pathological stress. Ryu et al.^94^ investigated the role of microgravity in regulating autophagy using GFP‐LC3 cells. The results suggested that AMPK was activated in response to microgravity‐induced cellular stress. These results suggest that prolonged exposure to microgravity can induce autophagy.^94^

## 6 EFFECT OF MICROGRAVITY ON CHEMOTHERAPY

Microgravity has been reported to induce significant alterations in cellular shape, size, volume and adherence properties of cancer cells.^95^ However, understanding the molecular mechanisms is crucial for developing potential strategies to modulate cancer cells.^95^ To date, there are limited studies carried out to understand the behaviour of cancer cells in response to chemotherapy under microgravity conditions. Recently, Prashanth et al.^1^ investigated the effect of microgravity on leukaemia cancer cells (HL40 and K562 cells) in response to chemotherapeutic agents (Doxorubicin and Daunorubicin). The findings demonstrate a notable influence of microgravity on cellular pathways, particularly ROS‐sensitivity pathways and an increase in the effectiveness of drugs. However, the impact of microgravity on cellular pharmacological responses appears to be predominantly influenced by the specific cell type.^1^

Cancer therapy involves the use of diverse drugs to target and eliminate cancer cells, however, the major challenge is the development of resistance to therapy due to the overexpression of multidrug resistance (MDR) proteins.^84^ A recent study by Rembiałkowska et al.^96^ investigated the effect of microgravity in combination with chemotherapy (Doxorubicin) on the chemoresistant (EPG85‐257 RDB) and sensitive (EPG85‐257 P) gastric cancer cells. The results showed microgravity combined with chemotherapy showed decreased expression of drug resistance‐related genes and increased marker in DNA/RNA damage.

## 7 DISCUSSION AND CONCLUSIONS

Physical forces such as gravity and electromagnetic forces influence a wide range of biological processes and contribute to the overall function, development and maintenance of organisms.

Microgravity alteration encompasses variation in cell membranes, cytoskeletal rearrangement, limited proliferation and shift in protein and expression of genes, involved in the synthesis of gravity‐sensing proteins, proteins involved in cell differentiation, migration and signalling pathways associated with autophagy. These intricate interplays between microgravity and different biomolecules play a key role in regulating cell behaviour.

Microgravity can be simulated on Earth using various simulation methods and using these methods, we can potentially harness the effect of microgravity in targeting cancer. Chemotherapy, a widely used treatment strategy against cancer metastasis, faces a major challenge associated with the development of resistance. Hence, researchers are keen on developing alternative strategies in combination with chemotherapy to mitigate resistance to cancer. In our study, based on the previous research, we investigated the interplay between microgravity and chemotherapy on cancer cells. The altered gravity demonstrated a modulatory effect on the expression of various genes related to cytoskeleton proteins, metabolic pathways and ROS pathways.

In conclusion, our results suggest the application of microgravity in cancer treatment, showcasing its potential to increase cell sensitivity to chemotherapy. These observed alterations in cancer cells in response to microgravity could be harnessed for developing a promising avenue for the development of a new therapeutic strategy against cancer. Hence, researchers are keen on developing a strategy by utilizing chemotherapy in combination with SMG to combat the growth and proliferation of cancer cells. Earlier investigations have shown encouraging outcomes in both cell line and animal studies by enhancing the responsiveness of cancer cells to chemotherapy. Further comprehensive exploration of these findings in human subjects holds promise for refining and advancing this innovative approach in cancer treatment.

## AUTHOR CONTRIBUTIONS

**Preksha Manish Vora:** Data curation (equal); methodology (equal); writing – original draft (equal); writing – review and editing (equal). **Sudharshan Prabhu:** Conceptualization (lead); funding acquisition (lead); project administration (lead); resources (lead); writing – original draft (lead); writing – review and editing (lead).

## FUNDING INFORMATION

This research was supported by the Indian Council of Medical Research (Sanction number. 54/8/GER/2019‐NCD‐II) and DBT‐BUILDER (BT/INF/22/SP43065/2021), Govt. of India. Manipal Research Board (MRB) Grant, and MAHE Seed Money Grant.

## CONFLICT OF INTEREST STATEMENT

The authors declare that the research was conducted in the absence of any commercial or financial relationships that could be construed as a potential conflict of interest.

## Data Availability

Data sharing is not applicable to this article, as no new data were created or analysed in this study.

## References

[1] Prasanth D , Suresh S , Prathivadhi‐Bhayankaram S , et al. Microgravity modulates effects of chemotherapeutic drugs on cancer cell migration. Life. 2020;10:162. 32846924 10.3390/life10090162 PMC7555236

[2] Bradbury P , Wu H , Choi JU , et al. Modeling the impact of microgravity at the cellular level: implications for human disease. Front Cell Dev Biol. 2020;8:96. 32154251 10.3389/fcell.2020.00096 PMC7047162

[3] Man J , Graham T , Squires‐Donelly G , Laslett AL . The effects of microgravity on bone structure and function. NPJ Microgravity. 2022;8:9. 35383182 10.1038/s41526-022-00194-8 PMC8983659

[4] Crucian BE , Choukèr A , Simpson RJ , et al. Immune system dysregulation during spaceflight: potential countermeasures for deep space exploration missions. Front Immunol. 2018;9:1437. 30018614 10.3389/fimmu.2018.01437 PMC6038331

[5] Bacci S , Bani D . The epidermis in microgravity and unloading conditions and their effects on wound healing. Front Bioeng Biotechnol. 2022;10:666434. 35392403 10.3389/fbioe.2022.666434 PMC8980714

[6] Collet P , Uebelhart D , Vico L , et al. Effects of 1‐ and 6‐month spaceflight on bone mass and biochemistry in two humans. Bone. 1997;20:547‐551. 9177869 10.1016/s8756-3282(97)00052-5

[7] Health (US), N. I. of, Study, B. S. C . Understanding Cancer. Biological Sciences Curriculum Study. NIH Curriculum Supplement Series. National Institutes of Health; 2007.

[8] Fares J , Fares MY , Khachfe HH , Salhab HA , Fares Y . Molecular principles of metastasis: a hallmark of cancer revisited. Signal Transduct Target Ther. 2020;5:28. 32296047 10.1038/s41392-020-0134-x PMC7067809

[9] Chang D , Xu H , Guo Y , et al. Simulated microgravity alters the metastatic potential of a human lung adenocarcinoma cell line. In Vitro Cell Dev Biol Ani. 2013;49:170‐177. 10.1007/s11626-013-9581-9 23404217

[10] Tan X , Xu A , Zhao T , et al. Simulated microgravity inhibits cell focal adhesions leading to reduced melanoma cell proliferation and metastasis via FAK/RhoA‐regulated mTORC1 and AMPK pathways. Sci Rep. 2018;8:3769. 29491429 10.1038/s41598-018-20459-1 PMC5830577

[11] Liu YN , Kang BB , Chen JH . Transcriptional regulation of human osteopontin promoter by C/EBPα and AML‐1 in metastatic cancer cells. Oncogene. 2004;23:278‐288. 14712233 10.1038/sj.onc.1207022

[12] Xin T , Greco V , Myung P . Hardwiring stem cell communication through tissue structure. Cell. 2016;164:1212‐1225. 26967287 10.1016/j.cell.2016.02.041 PMC4805424

[13] Ferranti F , Del Bianco M , Pacelli C . Advantages and limitations of current microgravity platforms for space biology research. Appl Sci. 2021;11:68.

[14] Grimm D , Egli M , Krüger M , et al. Tissue engineering under microgravity conditions‐ use of stem cells and specialized cells. Stem Cells Dev. 2018;27:787‐804. 29596037 10.1089/scd.2017.0242

[15] Mesland DA , Anton AH , Willemsen H , van den Ende H . The free fall machine‐ a ground‐based facility for microgravity research in life sciences. Microgravity Sci Technol. 1996;9:10‐14. 11539379

[16] Calvaruso M , Militello C , Minafra L , et al. Biological and mechanical characterization of the random positioning machine (RPM) for microgravity simulations. Life. 2021;11:1190. 34833068 10.3390/life11111190 PMC8619501

[17] Afonin BV . Analysis of possible causes activation a stomach and pancreas excretory and incretory function after completion of space flight on the international space station. Fiziol Cheloveka. 2013;39:62‐70. 25509873

[18] Hasenstein KH , Jack J , Loon J . Clinostats and other rotating systems‐design, function, and limitations. Gen Appl Extra‐Terr Environ On Earth. 2015;14:147‐156.

[19] Sarkar D , Nagaya T , Koga K , et al. Culture in vector‐averaged gravity under clinostat rotation results in apoptosis of osteoblastic ROS 17/2.8 cells. J Bone Miner Res. 2000;15:489‐498. 10750563 10.1359/jbmr.2000.15.3.489

[20] Chen ZY , Jiang N , Guo S , et al. Effect of simulated microgravity on metabolism of HGC‐27 gastric cancer cells. Oncol Lett. 2020;19:3439‐3450. 32269617 10.3892/ol.2020.11451 PMC7115135

[21] Brungs S , Egli M , Wuest SL , et al. Facilities for simulation of microgravity in the esa ground‐based facility programme. Microgravity Sci Technol. 2016;28:191‐203.

[22] Chen ZY , Guo S , Li BB , et al. Effect of weightlessness on the 3D structure formation and physiologic function of human cancer cells. Biomed Res Int. 2019;2019:4894083. 31073526 10.1155/2019/4894083 PMC6470427

[23] Mitteregger R , Vogt G , Rossmanith E , Falkenhagen D . Rotary cell culture system (RCCS): A new method for cultivating hepatocytes on microcarriers. Int J Artif Organs. 1999;22:816‐822. 10654878

[24] Herranz R , Larkin OJ , Dijkstra CE , et al. Microgravity simulation by diamagnetic levitation: effects of a strong gradient magnetic field on the transcriptional profile of *Drosophila melanogaster* . BMC Genomics. 2012;13:52. 22296880 10.1186/1471-2164-13-52 PMC3305489

[25] Valles JM , Maris HJ , Seidel GM , et al. Magnetic levitation‐based Martian and lunar gravity simulator. Adv Space Res. 2005;36:114‐118. 16252445 10.1016/j.asr.2005.01.081

[26] Chowdhury P , Long A , Harris G , Soulsby ME , Dobretsov M . Animal model of simulated microgravity: a comparative study of hindlimb unloading via tail versus pelvic suspension. Physiol Rep. 2013;1:e00012. 24303103 10.1002/phy2.12 PMC3831940

[27] Tesch PA , Lundberg TR , Fernandez‐Gonzalo R . Unilateral lower limb suspension: from subject selection to omic responses. J Appl Physiol. 2016;120:1207‐1214. 26846557 10.1152/japplphysiol.01052.2015

[28] Mortreux M , Rosa‐Caldwell ME . Approaching gravity as a continuum using the rat partial weight‐bearing model. Life. 2020;10:235. 33049988 10.3390/life10100235 PMC7599661

[29] Pandiarajan M , Hargens AR . Ground‐based analogs for human spaceflight. Front Physiol. 2020;11:716. 32655420 10.3389/fphys.2020.00716 PMC7324748

[30] Hargens AR , Vico L . Long‐duration bed rest as an analog to microgravity. J Appl Physiol. 2016;120:891‐903. 26893033 10.1152/japplphysiol.00935.2015

[31] Tomilovskaya E , Shigueva T , Sayenko D , Rukavishnikov I , Kozlovskaya I . Dry immersion as a ground‐based model of microgravity physiological effects. Front Physiol. 2019;10:284. 30971938 10.3389/fphys.2019.00284 PMC6446883

[32] Norsk P . Gravitational stress and volume regulation. Clin Physiol. 1992;12:505‐526. 1395444 10.1111/j.1475-097x.1992.tb00355.x

[33] Watenpaugh DE . Analogs of microgravity: head‐down tilt and water immersion. J Appl Physiol. 2016;120:904‐914. 26869710 10.1152/japplphysiol.00986.2015

[34] Adams GR , Caiozzo VJ , Baldwin KM . Skeletal muscle unweighting: spaceflight and ground‐based models. J Appl Physiol. 2003;95:2185‐2201. 14600160 10.1152/japplphysiol.00346.2003

[35] Bloomfield SA . Changes in musculoskeletal structure and function with prolonged bed rest. Med Sci Sports Exerc. 1997;29:197‐206. 9044223 10.1097/00005768-199702000-00006

[36] Gaponova AV , Rodin S , Mazina AA , Volchkov PV . Epithelial‐mesenchymal transition: role in cancer progression and the perspectives of antitumor treatment. Acta Naturae. 2020;12:4‐23. 10.32607/actanaturae.11010 PMC7604894 33173593

[37] Ratajczak MZ , Bujko K , Mack A , Kucia M , Ratajczak J . Cancer from the perspective of stem cells and misappropriated tissue regeneration mechanisms. Leukemia. 2018;32:2519‐2526. 30375490 10.1038/s41375-018-0294-7 PMC6286324

[38] Seyfried TN , Huysentruyt LC . On the origin of cancer metastasis. Crit Rev Oncog. 2013;18:43‐73. 23237552 10.1615/critrevoncog.v18.i1-2.40 PMC3597235

[39] Birkbak NJ , McGranahan N . Cancer genome evolutionary trajectories in metastasis. Cancer Cell. 2020;37:8‐19. 31935374 10.1016/j.ccell.2019.12.004

[40] Markowitz SD , Bertagnolli MM . Molecular basis of colorectal cancer. N Engl J Med. 2009;361:2449‐2460. 20018966 10.1056/NEJMra0804588 PMC2843693

[41] Quail DF , Joyce JA . Microenvironmental regulation of tumor progression and metastasis. Nat Med. 2013;1:1423‐1437. 10.1038/nm.3394 PMC3954707 24202395

[42] Egeblad M , Werb Z . New functions for the matrix metalloproteinases in cancer progression. Nat Rev Cancer. 2002;2:161‐174. 11990853 10.1038/nrc745

[43] Jayatilaka H , Tyle P , Chen JJ , et al. Synergistic IL‐6 and IL‐8 paracrine signalling pathway infers a strategy to inhibit tumour cell migration. Nat Commun. 2017;8:15584. 28548090 10.1038/ncomms15584 PMC5458548

[44] Carmeliet P . VEGF as a key mediator of angiogenesis in cancer. Oncology. 2005;69:4‐10. 16301830 10.1159/000088478

[45] Lugassy C , Escande JP . The haematogenous theory of metastasis: récamier did not propose it. Virchows Arch. 1997;431:431. 10.1007/s004280050113 9463580

[46] Gonzalez DM , Medici D . Signaling mechanisms of the epithelial‐mesenchymal transition. Sci Signal. 2014;7:8. 10.1126/scisignal.2005189 PMC4372086 25249658

[47] Arvelo F , Sojo F , Cotte C . Progression and metastasis. Ecancermedicalscience. 2016;10:617. 26913068 10.3332/ecancer.2016.617 PMC4754119

[48] Nurwidya F , Takahashi F , Murakami A , Takahashi K . Epithelial‐mesenchymal transition in drug resistance and metastasis of lung cancer. Cancer Res Treat. 2012;44:151‐156. 23091440 10.4143/crt.2012.44.3.151 PMC3467417

[49] Shang Y , Cai X , Fan D . Roles of epithelial‐mesenchymal transition in cancer drug resistance. Curr Cancer Drug Targets. 2013;13:915‐929. 24168191 10.2174/15680096113136660097

[50] Iwatsuki M , Mimori K , Yokobori T , et al. Epithelial‐mesenchymal transition in cancer development and its clinical significance. Cancer Sci. 2010;101:293‐299. 19961486 10.1111/j.1349-7006.2009.01419.x PMC11159985

[51] Urruticoechea A , Alemany R , Balart J , Villanueva A , Vinals F , Capella G . Recent advances in cancer therapy: an overview. Curr Pharm Des. 2010;16:3‐10. 20214614 10.2174/138161210789941847

[52] Longley D , Johnston P . Molecular mechanisms of drug resistance. J Pathol. 2005;205:275‐292. 15641020 10.1002/path.1706

[53] Mansoori B , Mohammadi A , Davudian S , Shirjang S , Baradaran B . The different mechanisms of cancer drug resistance: a brief review. Adv Pharm Bull. 2017;7:339‐348. 29071215 10.15171/apb.2017.041 PMC5651054

[54] Ramos A , Sadeghi S , Tabatabaeian H . Battling chemoresistance in cancer: root causes and strategies to uproot them. Int J Mol Sci. 2021;22:9451. 34502361 10.3390/ijms22179451 PMC8430957

[55] Koper K , Wileński S , Koper A . Advancements in cancer chemotherapy. Phys Sci Rev. 2023;8:583‐604.

[56] Gilman A . The initial clinical trial of nitrogen mustard. Am J Surg. 1963;105:574‐578. 13947966 10.1016/0002-9610(63)90232-0

[57] Abo‐Ghalia MH , Moustafa GO , Amr AE , et al. Anticancer activities of newly synthesized chiral macrocyclic heptapeptide candidates. Molecules. 2020;25:1253. 32164321 10.3390/molecules25051253 PMC7179445

[58] Eddy GS . Neoadjuvant chemotherapy before surgery in cervical cancer. J Natl Cancer Inst Monogr. 1996;21:93‐99. 9023836

[59] Schrag D , Cramer LD , Bach PB , Begg CB . Age and adjuvant chemotherapy use after surgery for stage III colon cancer. J Natl Cancer Inst. 2001;98:850‐857. 10.1093/jnci/93.11.850 11390534

[60] Malhotra V , Perry MC . Classical chemotherapy: mechanisms, toxicities and the therapeutc window. Cancer Biol Ther. 2003;2:1‐3. 14508075

[61] Lowenthal RM , Eaton K . Toxicity of chemotherapy. hematology/oncology. Clinics. 1996;10:967‐990. 10.1016/s0889-8588(05)70378-6 8811311

[62] Kim ES . Chemotherapy for lung cancer in the era of personalized medicine. J Thorac Oncol. 2010;5:219‐224.

[63] Monti N , Masiello MG , Proietti S , et al. Survival pathways are differently affected by microgravity in normal and cancerous breast cells. Int J Mol Sci. 2021;22:862. 33467082 10.3390/ijms22020862 PMC7829699

[64] Ong MS , Deng S , Halim CE , et al. Cytoskeletal proteins in cancer and intracellular stress: a therapeutic perspective. Cancer. 2020;12:238. 10.3390/cancers12010238 PMC7017214 31963677

[65] Wickstead B , Gull K . The evolution of the cytoskeleton. J Cell Biol. 2011;194:513‐525. 21859859 10.1083/jcb.201102065 PMC3160578

[66] Cogoli A . Signal transduction in T lymphocytes in microgravity. Gravit Space Biol Bull. 1997;10:5‐16. 11540120

[67] Hatton JP , Gaubert F , Lewis ML , et al. The kinetics of translocation and cellular quantity of protein kinase C in human leukocytes are modified during spaceflight. FASEB Journal. 1999;13:S23‐S33. 10352142 10.1096/fasebj.13.9001.s23

[68] Hammond TG , Benes E , O'Reilly KC , et al. Mechanical culture conditions effect gene expression: gravity‐induced changes on the space shuttle. Physiol Genomics. 2000;3:163‐173. 11015612 10.1152/physiolgenomics.2000.3.3.163

[69] Vorselen D , Roos WH , Mackintosh FC , Wuite GJL , Van Loon JJWA . The role of the cytoskeleton in sensing changes in gravity by nonspecialized cells. FASEB J. 2013;28:536‐547. 24249634 10.1096/fj.13-236356

[70] Guignandon A , Usson Y , Laroche N , et al. Effects of intermittent or continuous gravitational stresses on cell‐matrix adhesion: quantitative analysis of focal contacts in osteoblastic ROS. Exp Cell Res. 1997;236:66‐75. 9344586 10.1006/excr.1997.3703

[71] Medha M , Roy A . Microgravity: new aspect for breast cancer treatment, a review. Acta Astronaut. 2022;190:62‐67.

[72] Ingram M , Techy GB , Saroufeem R , et al. Three‐dimensional growth patterns of various human tumor cell lines in simulated microgravity of a NASA bioreactor. In Vitro Cell Dev Biol Ani. 1997;33:459‐466. 10.1007/s11626-997-0064-8 9201514

[73] Xu W , Mezencev R , Kim B , Wang L , McDonald JF , Sulchek T . Cell stiffness is a biomarker of the metastatic potential of ovarian cancer cells. PLoS One. 2012;7:e46609. 23056368 10.1371/journal.pone.0046609 PMC3464294

[74] Vassy J , Portet S , Beil M , et al. Effect of weightlessness on cytoskeleton architecture and proliferation of human breast cancer cell line MCF‐7. FASEB J. 2001;15:1104‐1106. 11292682 10.1096/fj.00-0527fje

[75] Duffy MJ . The role of proteolytic enzymes in cancer invasion and metastasis. Clin Exp Metastasis. 1992;10:145‐155. 1582084 10.1007/BF00132746

[76] Kurizaki T , Toi M , Tominaga T . Relationship between matrix metalloproteinase expression and tumor angiogenesis in human breast carcinoma. Oncol Rep. 1998;5:673‐677. 9538174 10.3892/or.5.3.673

[77] Dalberg K , Eriksson E , Enberg U , Kjellman M , Bäckdahl M . Membrane type 1 matrix metalloproteinase, and extracellular matrix metalloproteinase inducer mRNA expression: correlation with invasive growth of breast cancer. World J Surg. 2000;24:334‐340. 10658069 10.1007/s002689910053

[78] Hanemaaijer R , Verheijen JH , Maguire TM , et al. Increased gelatinase‐A and gelatinase‐B activities in malignant vs. benign breast tumors. Int J Cancer. 2000;86:204‐207. 10738247 10.1002/(sici)1097-0215(20000415)86:2<204::aid-ijc9>3.0.co;2-6

[79] Iwata H , Kobayashi S , Iwase H , Masaoka A , Fujimoto N , Okada Y . Production of matrix metalloproteinases and tissue inhibitors of metalloproteinases in human breast carcinomas. Jpn J Cancer Res. 1996;87:602‐611. 8766524 10.1111/j.1349-7006.1996.tb00266.x PMC5921148

[80] Liotta LA , Steeg PS , Stetler‐Stevenson WG . Cancer metastasis and angiogenesis: an imbalance of positive and negative regulation. Cell. 1991;64:327‐336. 1703045 10.1016/0092-8674(91)90642-c

[81] Ahn CB , Lee JH , Han DG , et al. Simulated microgravity with floating environment promotes migration of non‐small cell lung cancers. Sci Rep. 2019;9:14553. 31601869 10.1038/s41598-019-50736-6 PMC6787256

[82] Infanger M , Kossmehl P , Shakibaei M , et al. Simulated weightlessness changes the cytoskeleton and extracellular matrix proteins in papillary thyroid carcinoma cells. Cell Tissue Res. 2006;324:267‐277. 16432709 10.1007/s00441-005-0142-8

[83] Lebrun JJ . The dual role of TGFβ in human cancer: from tumor suppression to cancer metastasis. ISRN Mol Biol. 2012;2012:381428. 27340590 10.5402/2012/381428 PMC4899619

[84] Bukowski K , Kciuk M , Kontek R . Mechanisms of multidrug resistance in cancer chemotherapy. Int J Mol Sci. 2020;21:3233. 32370233 10.3390/ijms21093233 PMC7247559

[85] Corydon TJ , Kopp S , Wehland M , et al. Alterations of the cytoskeleton in human cells in space proved by life‐cell imaging. Sci Rep. 2016;6:20043. 26818711 10.1038/srep20043 PMC4730242

[86] Melnik D , Krüger M , Schulz H , et al. The CellBox‐2 mission to the international space station: thyroid cancer cells in space. Int J Mol Sci. 2021;22:8777. 34445479 10.3390/ijms22168777 PMC8395939

[87] Riwaldt S , Bauer J , Pietsch J , et al. The importance of Caveolin‐1 as key‐regulator of three‐dimensional growth in thyroid cancer cells cultured under real and simulated microgravity conditions. Int J Mol Sci. 2015;16:28296‐28310. 26633361 10.3390/ijms161226108 PMC4691055

[88] Agarwal E , Brattain MG , Chowdhury S . Cell survival and metastasis regulation by Akt signaling in colorectal cancer. Cell Signal. 2013;25:1711‐1719. 23603750 10.1016/j.cellsig.2013.03.025 PMC3686084

[89] Arun RP , Sivanesan D , Vidyasekar P , et al. PTEN/FOXO3/AKT pathway regulates cell death and mediates morphogenetic differentiation of colorectal cancer cells under simulated microgravity. Sci Rep. 2017;7:5952. 28729699 10.1038/s41598-017-06416-4 PMC5519599

[90] Vidyasekar P , Shyamsunder P , Arun R , et al. Genome‐wide expression profiling of cancer cell lines cultured in microgravity reveals significant dysregulation of cell cycle and microrna gene networks. PLoS One. 2015;10:e0135958. 26295583 10.1371/journal.pone.0135958 PMC4546578

[91] Jones CA , Hazlehurst LA . Role of calcium homeostasis in modulating EMT in cancer. Biomedicine. 2021;9:1200. 10.3390/biomedicines9091200 PMC8471317 34572386

[92] Fan RS , Jácamo RO , Jiang X , Sinnett‐Smith J , Rozengurt EG . Protein‐coupled receptor activation rapidly stimulates focal adhesion kinase phosphorylation at Ser‐843. Mediation by Ca^2+^, calmodulin, and Ca^2+^/calmodulin‐dependent kinase II. J Biol Chem. 2005;280:24212‐24220. 15845548 10.1074/jbc.M500716200

[93] Shi Z , Rao W , Wang H , et al. Modeled microgravity suppressed invasion and migration of human glioblastoma U87 cells through downregulating store‐operated calcium entry. Biochem Biophys Res Commun. 2015;457:378‐384. 25580009 10.1016/j.bbrc.2014.12.120

[94] Ryu HW , Choi SH , Namkoong S , et al. Simulated microgravity contributes to autophagy induction by regulating AMP‐activated protein kinase. DNA Cell Biol. 2014;33:128‐135. 24387300 10.1089/dna.2013.2089

[95] Buken C , Sahana J , Corydon TJ , et al. Morphological and molecular changes in juvenile normal human fibroblasts exposed to simulated microgravity. Sci Rep. 2019;9:11882. 31417174 10.1038/s41598-019-48378-9 PMC6695420

[96] Rembiałkowska N , Baczyńska D , Dubińska‐Magiera M , et al. RCCS bioreactor‐based modeled microgravity affects gastric cancer cells and improves the chemotherapeutic effect. Membranes. 2022;12:448. 35629774 10.3390/membranes12050448 PMC9146482

[97] Siddiqui IA , Sanna V , Ahmad N , et al. Resveratrol nanoformulation for cancer prevention and therapy. Annals of the New York Academy of Sciences. 2015;1348:20‐31. 26109073 10.1111/nyas.12811

